# Machine learning and spatio-temporal analysis of meteorological factors on waterborne diseases in Bangladesh

**DOI:** 10.1371/journal.pntd.0012800

**Published:** 2025-01-16

**Authors:** Arman Hossain Chowdhury, Md. Siddikur Rahman

**Affiliations:** Department of Statistics, Begum Rokeya University, Rangpur, Bangladesh; University of Oxford, UNITED KINGDOM OF GREAT BRITAIN AND NORTHERN IRELAND

## Abstract

**Background:**

Bangladesh is facing a formidable challenge in mitigating waterborne diseases risk exacerbated by climate change. However, a comprehensive understanding of the spatio-temporal dynamics of these diseases at the district level remains elusive. Therefore, this study aimed to fill this gap by investigating the spatio-temporal pattern and identifying the best tree-based ML models for determining the meteorological factors associated with waterborne diseases in Bangladesh.

**Methods:**

This study used district-level reported cases of waterborne diseases (cholera, amoebiasis, typhoid and hepatitis A) obtained from the Bangladesh Bureau of Statistics (BBS) and meteorological data (temperature, relative humidity, wind speed, and precipitation) sourced from NASA for the period spanning 2017 to 2020. Exploratory spatial analysis, spatial regression and tree-based machine learning models were utilized to analyze the data.

**Results:**

From 2017 and 2020, Bangladesh reported 73, 606 cholera, 38, 472 typhoid, 2, 510 hepatitis A and 1, 643 amoebiasis disease cases. Among the waterborne diseases cholera showed higher incidence rates in Chapai-Nawabganj (456.23), Brahmanbaria (417.44), Faridpur (225.07), Nilphamari (188.62) and Pirojpur (171.62) districts. The spatial regression model identified mean temperature (β = 12.16, s.e: 3.91) as the significant risk factor of waterborne diseases. The optimal XGBoost model highlighted mean and minimum temperature, relative humidity and precipitation as determinants associated with waterborne diseases in Bangladesh from 2017 to 2020.

**Conclusions:**

The findings from the study, incorporating the One Health perspective, provide insights for planning early warning, prevention, and control strategies to combat waterborne diseases in Bangladesh and similar endemic countries. Precautionary measures and intensified surveillance need to be implemented in certain high-risk districts for waterborne diseases across the country.

## Author summary

Bangladesh is increasingly vulnerable to waterborne illnesses, exacerbated by climate change. This study uses spatial analysis, regression, and machine learning models to investigate the spatiotemporal patterns of cholera, typhoid, hepatitis A, and amoebiasis across districts from 2017 to 2020. Districts with the highest prevalence of cholera include Brahmanbaria, Chapai-Nawabganj, and others. The study identifies mean and minimum temperatures, relative humidity, and precipitation as the main climatic factors influencing disease transmission. The findings highlight the need for targeted interventions, early warning systems, and improved surveillance in high-risk areas to prevent waterborne infections. These insights align with the One Health approach to managing health risks in Bangladesh and similar regions.

## Introduction

Waterborne diseases (WBDs) are illnesses brought on by harmful microorganisms that are spread by water, including bacteria, viruses, and protozoa. These microorganisms might have detrimental impacts on human health, including disability, disease, disorders, or death, if action is delayed [1]. When contaminated water is used for drinking, cooking, or cleaning clothing, these germs can spread [2]. However, the majority of waterborne diseases are transmitted by the fecal–oral route, which is mainly brought on by improper management of waste and sanitation. This pathway occurs when human or animal feces, such as those from rats, are consumed by drinking polluted water or eating contaminated food. Waterborne pathogens cause death and disability, significantly impacting public health and accelerating the onset of waterborne illnesses [3]. WBDs include cholera, amoebiasis, typhoid, hepatitis A etc. WBDs cause 2.2 million fatalities annually worldwide, as more than 2.1 billion individuals lack access to safe drinking water [4]. According to the World Health Organization (WHO), as of 2019, an estimated 9 million people fall ill with typhoid annually, and approximately 110,000 people die from the disease each year [5].

Bangladesh faces significant risk of contracting waterborne diseases [6] because of several issues, including persistent climate, inadequate sanitation, overpopulation, lack of pure water access, and scarcity of medical resources [7,8]. Historically, over the past 30 years, the average annual temperature in Bangladesh hovers around 26°C, with seasonal fluctuations ranging between 15°C and 34°C [9,10]. Elevated temperatures could potentially foster the proliferation of waterborne diseases, making the northern and northwestern regions of the country particularly vulnerable. Additionally, it’s crucial to note that the extension of summers, milder winters, and unusually unpredictable monsoons may all influence the prevalence and transmission of these ailments [8]. Bangladesh is particularly susceptible to WBDs because of its geographical location, weather, and high population density [11], frequent flood [12] and rising sea levels [13]. The most prevalent waterborne infections in Bangladesh include cholera, typhoid fever, amoebiasis and hepatitis A. According to the Directorate General of Health Services (DGHS), more than 3,400 individuals have contracted different WBDs as a result of the floods since June 18, 2022 [14]. Prior studies has shown that meteorological factors such as temperature, relative humidity and precipitation impact the transmission of WBDs [15–17]. Studies have also indicated that these climatic factors, along with wind speed significantly affect tuberculosis [18,19]. Higher humidity was positively associated with malaria and diarrhea [20]. Additionally, the high population density [21], and weak healthcare infrastructure further exacerbate Bangladesh susceptible to waterborne diseases [22].

Several prior studies have employed various methods to explore the relationship between meteorological factors and different waterborne diseases, including time series analysis [23], the SEIAR model [24], Poisson regression model [25], lag non-linear model [26] and boosted regression tree model [27]. However, these studies investigated the relationship as a whole and didn’t capture the spatial characteristics of the diseases. While some research in Bangladesh has examined the link between meteorological factors and waterborne diseases [20,28], spatial analyses are lacking. For example, some studies [29,30] have begun exploring these patterns, but a comprehensive spatial analysis is needed to identify the most vulnerable areas and contributing meteorological factors. On the other hand, the transmission of waterborne disease is usually influenced by various factors which exhibit a nonlinear pattern that causes several issues. These issues can be effectively addressed by robust machine learning (ML) techniques, which handle nonlinear relationships through methods like feature transformation, ensemble approaches etc. ML models have proven to be highly robust and efficient for prediction and classification across a wide range of fields, including both communicable and non-communicable diseases [17,31–35]. However, their potential remains largely untapped in the realm of waterborne diseases, particularly when it comes to analyzing continuous data. Therefore, the objective of our study was two-fold: first, to analyze the spatio-temporal patterns of different waterborne diseases using geospatial mapping to show incidence rates, and second, to apply spatial regression and the best tree-based ML models to pinpoint the crucial climate factors influencing these diseases in Bangladesh. The insights gained will help policymakers and government officials allocate resources to the most affected areas, enabling more targeted and effective interventions. This will support the development of early warning systems, preventive strategies, and control measures to address waterborne diseases and reduce their impact.

## Materials and methods

### Study location

**Fig 1:**
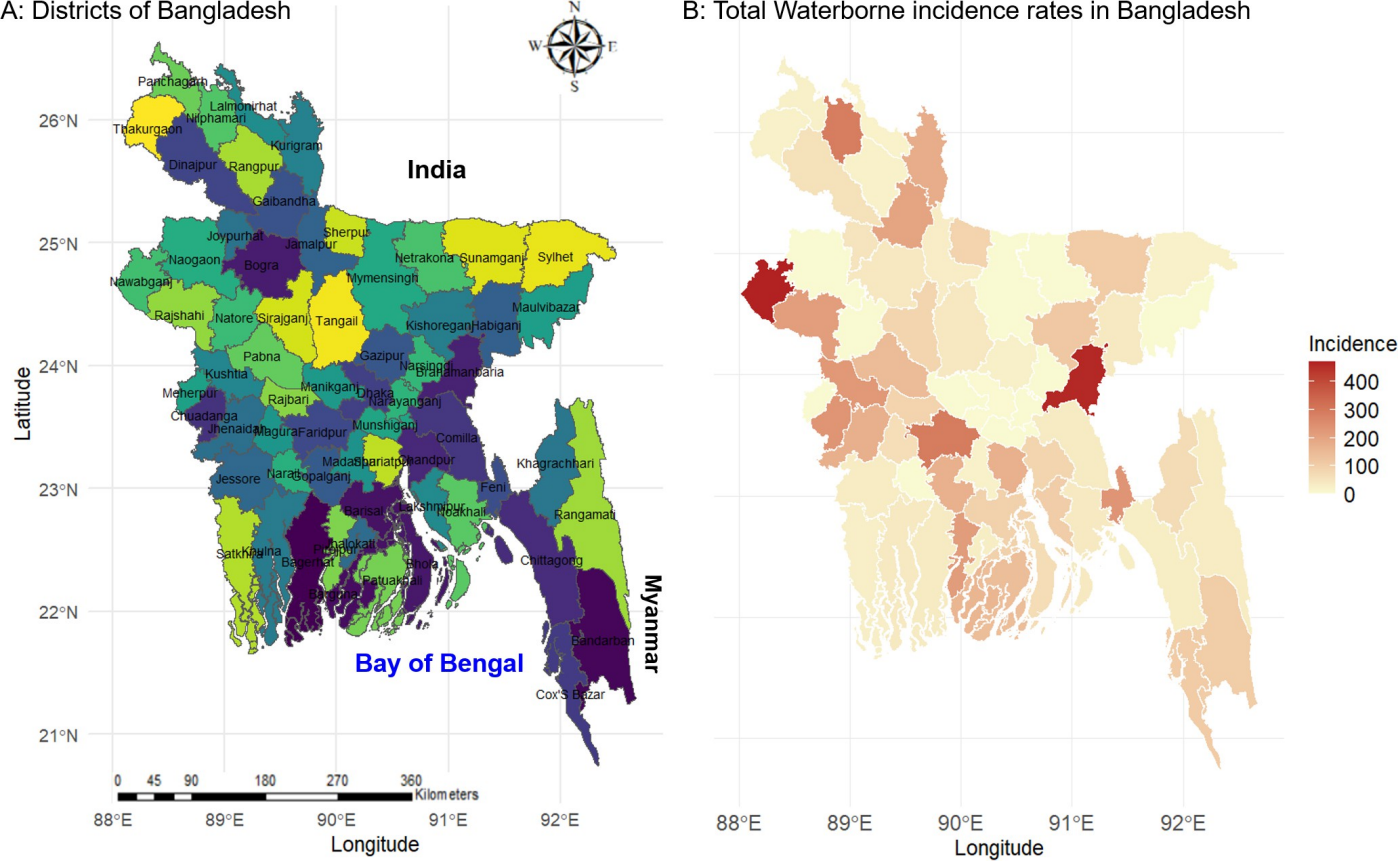
(A) Geographic map of Bangladesh showing its districts and neighboring countries, including the Bay of Bengal, (B) District-wise incidence rates of waterborne diseases per 100,000 individuals. For map creation, we utilized the ’ggplot2’, ’maps’, and ’sf’ packages with publicly available shapefile data sourced from Global Administrative Areas Database (GADM) [46].

Bangladesh, located in South Asia, spans latitudes between 20°34’ to 26°38’ north and longitudes between 88°01’ to 92°41’ east. It stretches approximately 440 km from east to west and 760 km from north-northwest to south-southeast [36]. The country covers a total area of 147,570 square kilometers and is divided into 64 districts across 8 divisions, all of which were subject to investigation in this study (Fig 1A) [37]. Due to its subtropical to tropical monsoon climate, Bangladesh undergoes pronounced seasonal shifts marked by significant rainfall, hot temperatures, and elevated humidity levels.

### Data source

Our study compiled a dataset of waterborne diseases—including cholera, amoebiasis, typhoid, and hepatitis A—and seven meteorological factors (maximum, minimum, and mean temperature, relative humidity, maximum and minimum wind speed, and precipitation) spanning from 2017 and 2020. The meteorological data were collected in yearly format from the NASA Langley Research Center (LaRC) website [38]. To ensure district-level data accuracy, the data were acquired using the latitude and longitude values corresponding to each district. Instead of using measurements from weather stations, the NASA data are reanalyzed grid data with a spatial resolution of ½° × ⅝° [39] that provide consistent geographic coverage appropriate for regional studies. One of the primary challenges in studying waterborne diseases in Bangladesh is data availability and granularity. The available waterborne disease data consist of yearly aggregated counts, with each data point representing the total number of recorded cases for a specific district and disease in a given year. These counts reflect the total number of infected individuals, derived from individual records. The disease cases (2017–20) were collected from Bangladesh Environment Statistics 2020, under the Strengthening Environment, Climate Change and Disaster Statistics (ECDS) Project. This project was carried out by the Department of Statistics and Information, Bangladesh Bureau of Statistics (BBS) [40]. To address missing values, we used Microsoft Excel (Version 2013) [41], applying the mean imputation method [42]. The rationale for employing mean imputation stems from its ease of use and ability to substitute missing values based on data distribution [43]. Subsequently, we performed a log transformation to mitigate issues related to outliers, skewness, and multicollinearity, which helped improve model fit. The weather factors were aligned with district-level health outcomes by matching the meteorological data with the corresponding district and year. The district-wise population data for computing incidence rate were obtained from the Population and Housing Census (PHC-2011) [44]. A detailed description of the data is presented in Table 1.

**Table 1:** Description of all input predictors and response variables.

Type	Codes	Description
Spatial temporal	Year	Year
	District	District names
	Latitude	Latitude values
	Longitude	Longitude values
Climate	x1	MERRA-2 Temperature at 2 Meters (C)
	x2	MERRA-2 Relative Humidity at 2 Meters (%)
	x3	MERRA-2 Temperature at 2 Meters Maximum (C)
	x4	MERRA-2 Temperature at 2 Meters Minimum (C)
	x5	MERRA-2 Precipitation Corrected (mm)
	x6	MERRA-2 Wind speed at 50 Meters Maximum (C)
	x7	MERRA-2 Wind speed at 50 Meters Minimum (C)
Waterborne diseases	y1	Cholera
	y2	Typhoid and Paratyphoid fevers
	y3	Amoebiasis
	y4	Acute Hepatitis A
	y5	Total Waterborne diseases

MERRA-2: Modern-Era Retrospective Analysis for Research and Applications, version 2

### Statistical analyses

**Fig 2:**
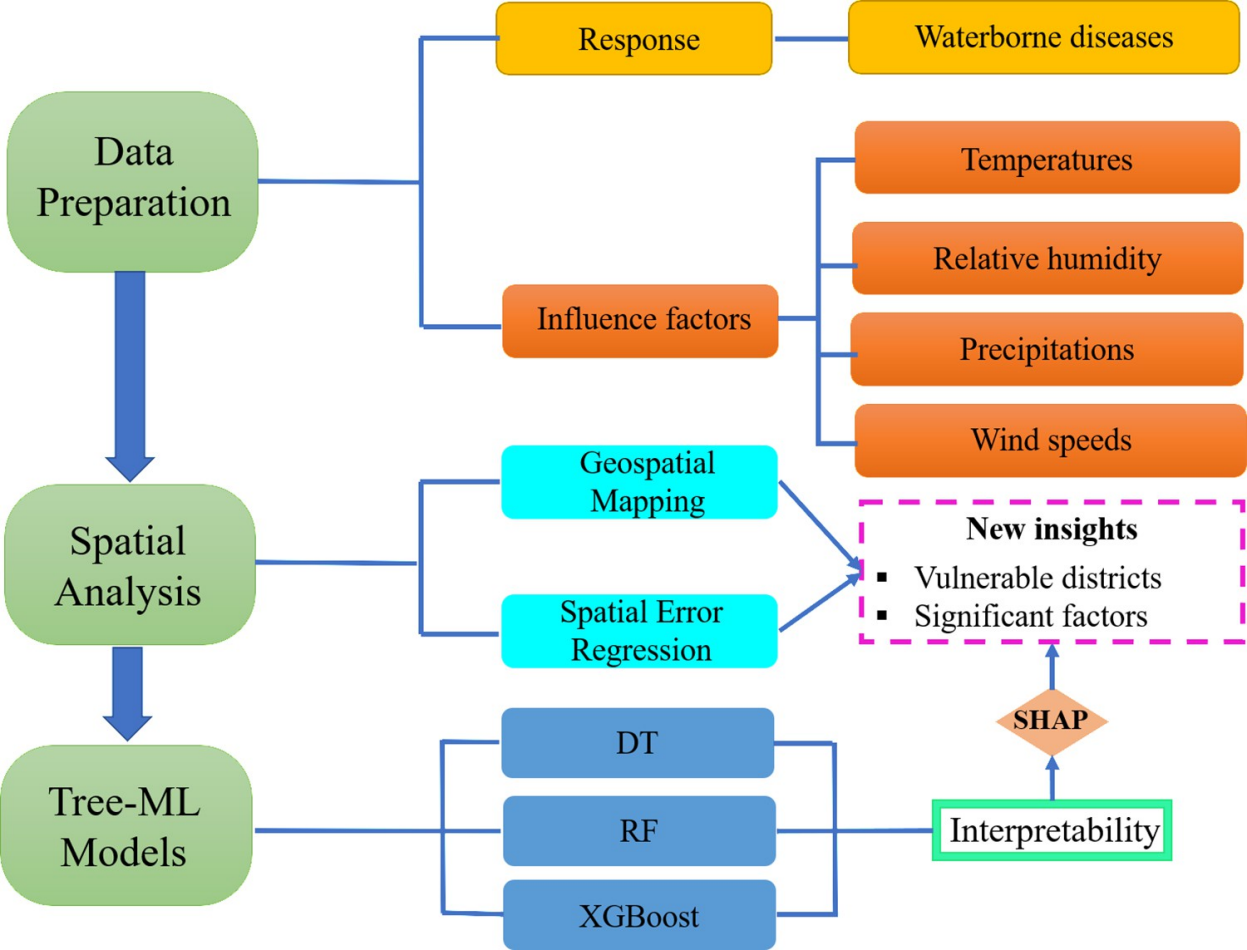
Overview of the proposed study design, including its key components. RF: Random Forest; DT: decision tree; XGBoost: eXtreme Gradient Boosting; SHAP: Shapely Additive eXplanation.

To calculate the incidence rates, we aggregated data for each disease across all districts and computed the incidence rate per 100,000 population (S1 Text) [36]. Using the transformed dataset, we conducted Pearson’s bivariate product-moment correlation analysis to initially assess the relationship between waterborne diseases and climate factors. To further investigate the impact of climate factors on waterborne diseases, we employed a spatial error regression model (Fig 2). We also evaluated the performance of three tree-based ML models to identify the best one for determining the meteorological risk factors associated with waterborne diseases. For developing the machine learning (ML) models in predicting waterborne diseases, we split the data into training and testing sets, with 70% of the data allocated for training and 30% for testing. All analyses, including spatial plots, correlation plots, spatial regression, and ML modeling, were performed using RStudio (Version 4.4.0) [45]. For map creation, we utilized the ’ggplot2’, ’maps’, and ’sf’ packages with publicly available shapefile data sourced from Global Administrative Areas Database (GADM) [46], while the correlation plot was generated using the ’ggcorrplot’ package. The spatial error regression model was constructed with the ’sp’, ’spData’, ’spdep’, and ’spatialreg’ packages. Furthermore, tree-based interpretable machine learning models were developed using a variety of R packages, such as ’caret’, ’xgboost’, ’dplyr’, ’MLmetrics’, ‘randomForest’, ‘rpart’ and others. We also conducted SHAP analysis using the ’SHAPforxgboost’ package. Data and detailed R codes for data analysis are available at https://github.com/arman2018/waterborne-disease-in-Bangladesh-from-2017-2020.

### Spatial error regression

The ordinary least squares (OLS) estimator, commonly used in linear regression models, was found to be less likely than other estimators to be the Best Linear Unbiased Estimator (BLUE) when there are geographical dependencies. When variable values show connections between geographic units, these dependencies become apparent [47]. Anselin (2002) proposed the spatial regression approach, which was employed to offer a more accurate estimation in situations where geographic dependencies were present.

The spatial error model was employed when the error term of the OLS model exhibited geographical dependencies. It encompassed the spatial error term (*W*_*ε*_), defined mathematically as follows:

$$y=X\beta +\varepsilon ,\varepsilon =\lambda W_{\varepsilon }+\mu$$ (1)

Where, y represents the response variable, representing waterborne diseases across districts (*n*×1 matrix), X represents the predictor variable which includes meteorological factors (*n*×*k* matrix), *W*_*ε*_ represents the spatial weight matrix, indicating spatial relationship between the districts (*n*×*n*), *λ* denotes the spatial error parameter measuring the strength of the spatial dependency in the error term, *β* is the slope of the regression (*k*×1) and *μ* represents the matrix of random error [48]. The widely used technique of moments was applied to estimate the spatial error model.

### Decision Tree (DT)

A tridiagonal (DT) methodology is one of the easiest and most natural methods in machine learning [49,50]. A DT allocates a class label (or outcome) to an input feature by classifying it using the tests in the tree, which have leaf nodes that are classifications of structures and interior nodes that are tests on input vectors. The results of each test are mutually exclusive and exhaustive in nature [51]. DTs play a key role in environmental epidemiology because they can simulate intricate interactions between environmental conditions and health outcomes, including the ability to forecast disease prevalence based on climatic variables [52]. Because of its hierarchical decision-making process, decision trees (DTs) are effective in modeling the association between climate parameters and the occurrence of waterborne diseases. DTs are flexible for both continuous and categorical data analysis, as they can be used for regression in addition to classification. In our study, we used DT as a regressor to analyze continuous waterborne disease data.

### Random Forest (RF)

A machine learning based classifier using decision trees is called random forest (RF), a method initially suggested by Breiman [53]. It can be performed in both classification and regression tasks and has been employed in a variety of biomedical studies [54,55]. RF is particularly valuable in environmental epidemiology because it can simulate intricate, non-linear relationships between various environmental components and health outcomes [56]. This makes it an effective technique for identifying the key factors that predict illnesses influenced by environmental factors such as air quality or climatic variables. Beyond its versatility, RF can handle both categorical and continuous data, allowing it to predict continuous outcomes like disease incidence rates. In this study, we used RF as a regressor to examine continuous data on waterborne diseases. We produced several trees which consist of a forest and voted for specified input variables using each tree in the forest. After computing the mean votes, RF provides a final prediction that is more robust and accurate [57]. The general equation of RF can be expressed as

$$Y=\frac{1}{P}\sum_{i=1}^{P}F_{i}(x)$$ (2)

Where, *Y* is the predicted disease cases, P denotes the number of trees in the ensemble and *F*_*i*_(*x*) is the output of the *i*^*th*^ tree for the input feature vector *x* (meteorological factors). In order to reduce variation and improve model performance, Random Forests (RF) were utilized in this work to simulate the association between climatic conditions and the prevalence of waterborne diseases. By averaging the findings across many decision trees, RF can identify the most relevant risk factors.

### XGBoost model

The eXtreme Gradient Boosting (XGBoost) is a tree-based ensemble ML technique that can increase the accuracy and strength of overall training and prediction by including several weak learners [35]. It was first developed in 2011 by Chen Tianqi and Carlos Gestrin, and in the subsequent study, several researchers refined and enhanced it [58]. It has demonstrated to be an effective and capable problem solution for machine learning, particularly in environmental epidemiology. It is valuable for both classification and regression for modeling non-linear relationships between environmental factors and health outcomes [59]. The main idea of boosting, which is the process of improving machine learning models, is to combine a large number of weak forecasting models into a single, robust ensemble model. Different models need to be frequently merged to have excellent prediction accuracy with acceptable parameter values. The model might need to be run several times or more in order to achieve appropriate precision for complex data. The XGBoost model can better handle this issue [60]. The general objective function of the XGBoost model is

$$Obj^{\left(t\right)}=\sum_{i=1}^{n}l(y_{i},{\hat{y}}_{i}^{\left(t-1\right)}+f_{t}(x_{i}))+\Omega (f_{t})+constant$$ (3)

Where *y*_*i*_ is the observed counts of disease cases, ${\hat{y}}_{i}^{\left(t-1\right)}$ indicates the predicted value from the previous iteration, *x*_*i*_ is the input vector of meteorological factors, n denotes the number of observations (district-level data points), *f*_*t*_ denotes a distinct function which algorithm trains, *Ω*(*f*_*t*_) is the regularization term which prevents models from overfitting. *l* represents the loss function, which computes the deviance between the label and the estimate in the earlier stage, the new tree’s output [17]. In this study, we employed XGBoost as a regressor to explore the relationship between meteorological variables and waterborne disease prevalence.

### Interpretation of machine learning models: SHAP (SHapley Additive eXplanations)

SHAP (SHapley Additive eXplanations) is a method for interpreting ML model output developed by Lundberg and Lee [61]. The term "Shapley Additive Explanation" refers to an additive explanation model developed by SHAP that was motivated by collaborative game theory and considered all attributes as “contributors”. The model creates a projected value for each estimated sample, and the SHAP value is the weighted average of all the features in the estimated sample. Consider an XGBoost model that predicts an output (N) from a group N (with n characteristics). According to each characteristic’s marginal impact the impact of each feature (∅_*i*_ is impact of feature i) on the model output v(N) is assigned in SHAP. Depending on a number of axioms to assist equitably distribute each feature’s influence, shapely values can be expressed by the following equation [62]:

$$\emptyset_{i}=\sum_{S\epsilon N}\frac{|S|!(n-|S|-1)!}{n!}[v(S\cup \{i\}-v(S)]$$ (4)

In our study, SHAP was employed to determine feature importance specifically for the best-performing ML model, identified through comparisons among the three models used, helping us interpret the contributions of various climate factors to the model’s predictions.

### Model validation and assessment metrics

The tree-based ML models were built using the training data, with hyperparameter tuning (S3 Table, S1–S3 Figs) and cross-validation to enhance model performance. Specifically, we utilized 10-fold cross-validation, a technique that divides the data into 10 subsets, iteratively training the model on 9 subsets while testing on the remaining one. This helps in reducing overfitting and underfitting [63]. Additionally, we applied L1 and L2 regularization [63] to further stabilize the models.

The primary assessment metric for model evaluation is the accuracy computation of model. The accuracy of the model refers to the closeness of the true and estimated values. There are numerous ways to determine the model’s accuracy. In our study, we utilized three distinct model accuracy metrics including mean absolute percentage error (MAPE), mean absolute error (MAE), and root mean square error (RMSE). These metrics can be explained mathematically as follows:

$$MAE=\frac{1}{n}\sum_{i=1}^{n}\left|\hat{y_{i}}-y_{i}\right|$$ (5)

$$RMSE=\sqrt{\frac{1}{n}\sum_{i=1}^{n}{\left(\hat{y_{i}}-y_{i}\right)}^{2}}$$ (6)

$$MAPE=\frac{1}{n}\sum_{i=1}^{n}|\frac{\hat{y_{i}}-y_{i}}{y_{i}}|\times 100\%$$ (7)

Where n denotes the number of observation, ${\hat{y}}_{i}$ denotes the estimated number and *y*_*i*_ represents the true number, and ${\hat{y}}_{i}-y_{i}$ represents the residual number [60].

## Results

### Characteristics of waterborne diseases

In our study, we examined four waterborne diseases. Among the waterborne diseases, cholera emerged as the most widespread, reaching its peak in 2017 and hitting a low point in 2020 (Table 2). Typhoid followed as the second most prevalent, with the highest occurrence in 2019 and the lowest in 2020. Meanwhile, amoebiasis, although less common, saw its highest incidence in 2017 and its lowest in 2020 in Bangladesh (Table 2).

The mean number of cholera cases varied less across the years, ranging from 147.05 to 388.18, with the highest mean of 388.18 cases recorded in 2017 (Table 2). Similarly, the mean number of amoebiasis cases varied from 2.09 to 10.20, with the highest mean number of 10.20 cases in 2017. More details about the summary statistics of the waterborne diseases are presented in Table 2.

**Table 2:** Descriptive statistics for different waterborne diseases in Bangladesh from 2017 to 2020.

Diseases	Year	Min	Median	Max	Mean±SD	Total
**Cholera**	2017	0	75	7576	388.14±1017.25	24841
	2018	0	74	3784	312.95±604.56	20029
	2019	0	45.50	6596	301.95±873	19325
	2020	0	25.50	1870	147.05±314.34	9411
**Typhoid**	2017	0	85	1131	175.73±242.12	11247
	2018	0	72.50	1217	156.14±223.11	9993
	2019	0	96.50	1231	196.25±265.19	12560
	2020	0	28	730	73±120.20	4672
**Amoebiasis**	2017	0	1	335	10.20±42.56	653
	2018	0	2	82	5.61±12.03	359
	2019	0	1	190	7.77±25.07	497
	2020	0	0	19	2.09±4.28	134
**Hepatitis A**	2017	0	3	64	6.80±10.93	435
	2018	0	6	212	15.45±30.54	989
	2019	0	6	158	11.31±20.93	724
	2020	0	2	67	5.75±9.94	362

Min: Minimum; Max: Maximum; SD: Standard deviation

### Characteristics of climate variables

**Fig 3:**
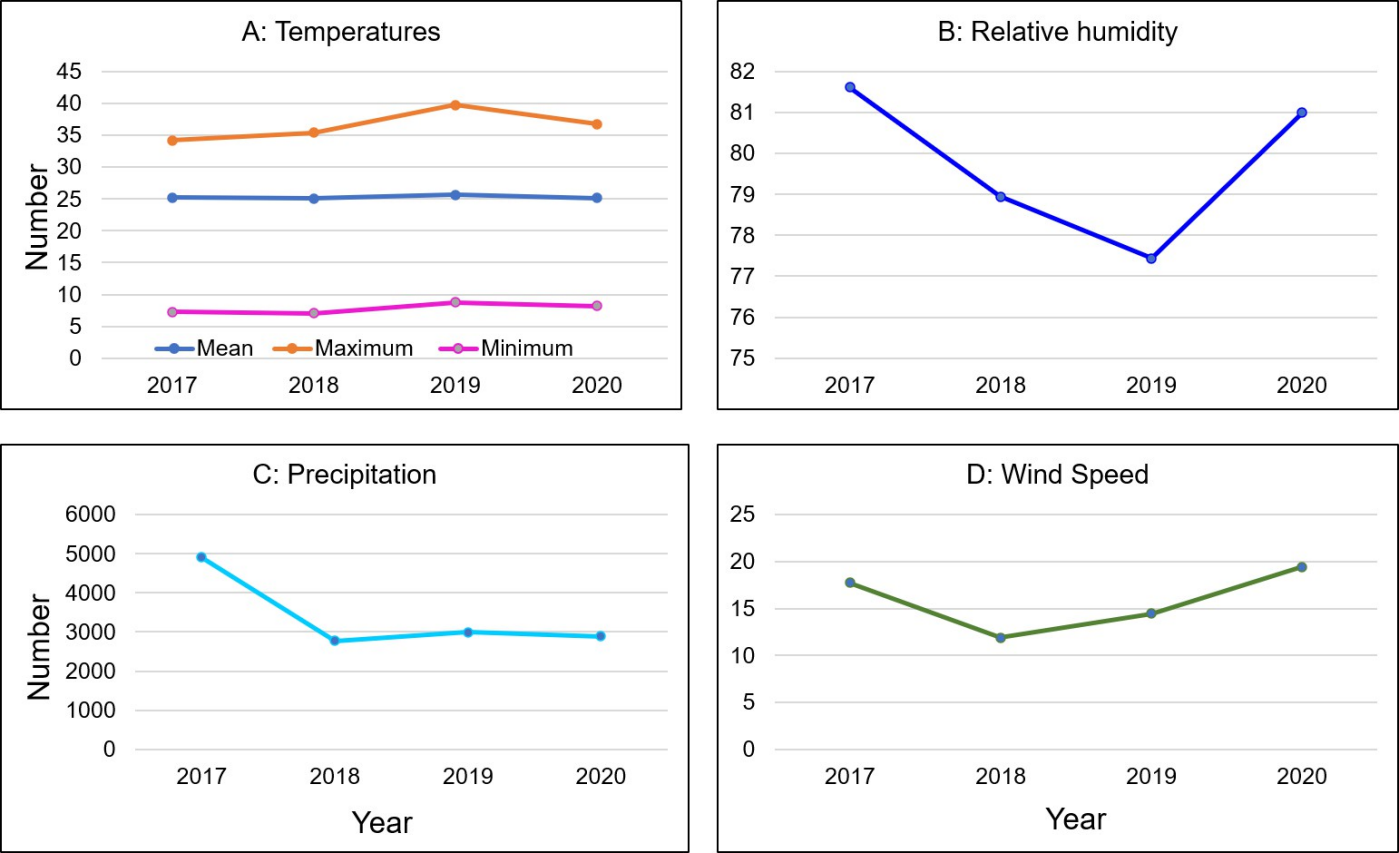
Time series plot of yearly climate factors from 2017 to 2020.

We included seven climate factors in the study including maximum, minimum, and mean temperature, relative humidity, maximum and minimum wind speed, and precipitation. Fig 3 depicts the temporal development of climatic factors from 2017 to 2020. The observed aberrations, peaks, and oscillations in the plot underscore the inherent nonlinear relationships within the data. Fluctuations in yearly mean, maximum and minimum temperatures, relative humidity, maximum and minimum wind speed, and precipitation levels resist a linear pattern, emphasizing a nonlinear pattern (Fig 3). The summary statistics of the climatic parameters will be found in S2 Table.

### Spatio-temporal pattern of waterborne diseases

**Fig 4:**
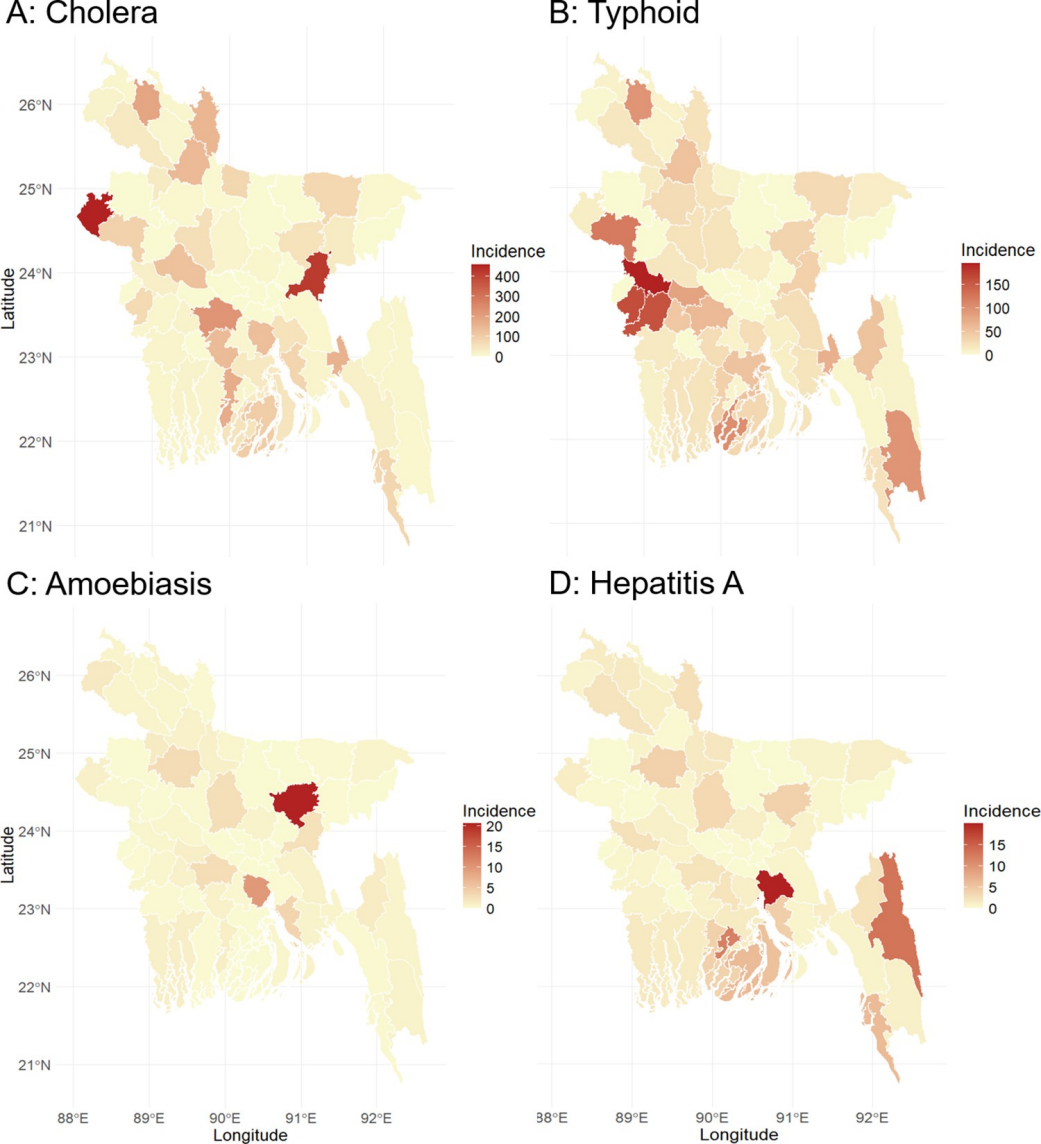
Spatial distribution of waterborne diseases incidence rates per 100,000 population in Bangladesh from 2017 to 2020. For map creation, we utilized the ’ggplot2’, ’maps’, and ’sf’ packages with publicly available shapefile data sourced from Global Administrative Areas Database (GADM) [46].

Waterborne diseases are more prevalent in Bangladeshi districts such as Chapai Nawabganj, Brahmanbaria, Faridpur, Nilphamari and Chuadanga (Fig 1B). Among these diseases, Cholera is the most prevalent waterborne disease in the Chapai Nawabganj, Brahmanbaria, Faridpur, Nilphamari and Pirojpur districts, whereas the central Bangladeshi districts of Manikganj, Munshiganj and many more districts reported no incidence of cholera (S1 Table). Typhoid is another highly prevalent disease in the Kushtia Chuadanga, Jhenaidah, Rajshahi and Barguna districts, with no incidence in Mymensingh, Netrokona and many more districts. Kishoreganj, Shariatpur, Laksmipur, Bogra and Faridpur districts reported the highest prevalence rate of amoebiasis, while Narayanganj, Narshingdi, Meherpur and many more reported no incidences of amoebiasis. Hepatitis A is more prevalent in Chandpur, Rangamati, Jhalokati, Patuakhali and Cox’s Bazar districts, whereas Maulvi bazar, Narail, Meherpur and many more districts reported no incidence of hepatitis A (Fig 4).

### Association of climate factors with waterborne diseases

**Fig 5:**
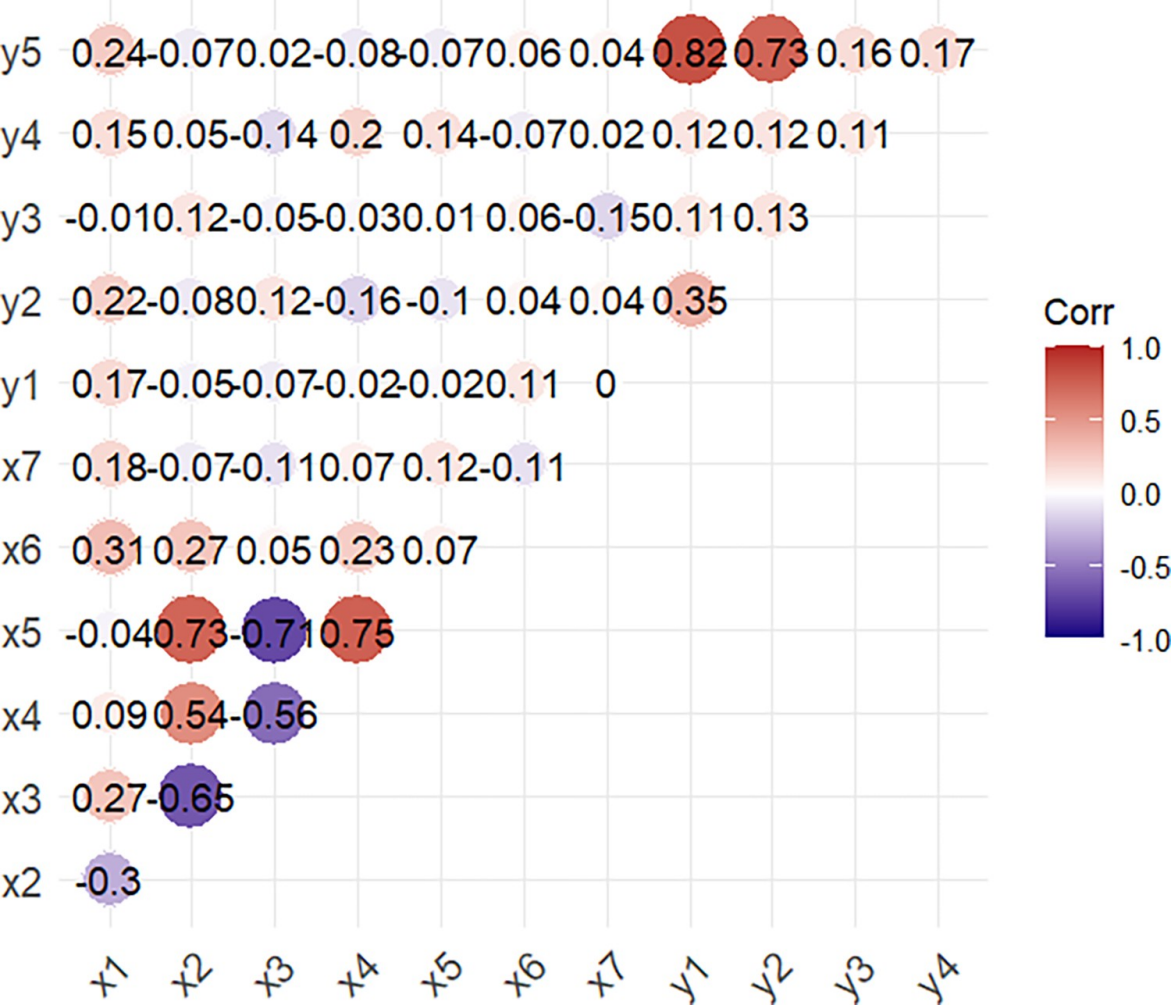
Pairwise correlation matrix illustrating the relationships between waterborne diseases and climate variables; x1: mean temperature; x2: relative humidity; x3: maximum temperature; x4: minimum temperature; x5: precipitation; x6: maximum wind speed; x7: minimum wind speed.

In the case of waterborne diseases, bivariate correlation analysis revealed that cholera was significantly associated with mean temperature (S4 Table). Mean and minimum temperatures were significantly associated with typhoid disease. Amoebiasis was significantly associated with relative humidity and minimum wind speed. Hepatitis A was significantly associated with mean, maximum, and minimum temperatures, as well as precipitation. Overall, we found that mean temperature was significantly correlated with the total cases of waterborne diseases (Fig 5).

The aforementioned significant climate factors were then used as covariates in the spatial error regression model, with each outcome variable. For example, in the first model, focusing on cholera disease, the spatial distribution of the residual was observed to exhibit a statistically significant positive association. The prevalence of cholera disease showed a positive association with mean temperature. In model 2, focusing on typhoid disease, the spatial distribution of the residual was observed to exhibit a statistically significant positive association. The prevalence of typhoid disease positively correlated with mean temperature but was negatively related to minimum temperature. In model 3, focusing on amoebiasis disease, the spatial distribution of the residual was observed to exhibit a statistically significant positive association. The prevalence of amoebiasis disease was negatively associated with maximum wind speed. In model 4, focusing on hepatitis A disease, the spatial distribution of the residual was observed to exhibit a statistically significant positive association. The prevalence of hepatitis A was positively associated with mean temperature but negatively associated with maximum temperature. In model 5, focusing on overall waterborne disease, the spatial distribution of the residual was observed to exhibit a statistically significant positive association. Waterborne illnesses were positively associated with mean temperature (Table 3).

**Table 3:** Estimated parameters of significant climate factors of spatial error model for different waterborne diseases.

Factors	Cholera Model 1		Typhoid Model 2		Amoebiasis Model 3		Hepatitis A Model 4		Overall Model 5	
	*Coef*	*s*.*e*	*Coef*	*s*.*e*	*Coef*	*s*.*e*	*Coef*	*s*.*e*	*Coef*	*s*.*e*
**Maximum Temperature**	–	–	–	–	–	–	-2.14*	1.13	–	–
**Minimum Temperature**	–	–	-1.25*	0.36	–	–	–	–	–	–
**Mean Temperature**	10.05*	5.65	21.45*	4.24	–	–	8.88*	3.78	12.16*	3.91
**Relative Humidity**	–	–	–	–	–	–	–	–		
**Minimum Wind Speed**	–	–	–	–	-0.18*	0.09	–	–	–	–
**Spatial error parameter (λ)**	0.44*	0.05	0.55*	0.04	0.28*	0.10	0.38*	0.05	0.40*	0.09

Coef: Coefficient; s.e: Standard error; Asterisk (*) indicates significance at 5% level

### Performance evaluation of ML models

The spatial regression model extends beyond the linear regression model and is utilized to identify linear relationships. However, the transmission of waterborne diseases is often influenced by various climatic factors exhibiting nonlinear patterns (Fig 3), posing challenges for linear models. This issue can be effectively addressed through ML techniques. In this study, the three tree-based ML models (DT, RF and XGBoost) were fitted and their performance was presented in Table 4. The assessed performance revealed that the XGBoost model is more efficient than DT and RF in predicting waterborne diseases in Bangladesh. For instance, the mean absolute percentage error (MAPE) values for the testing set of the XGBoost model were lower compared to the RF and DT models, with MAPE values of 0.13%.

**Table 4:** Performance evaluation with different metrics of the tree-based models that predict waterborne diseases in Bangladesh from 2017 to 2020.

Models	Dataset	RMSE	MAE	MAPE
**Decision Tree**	*Training*	898.44	446.33	0.80
	*Testing*	465.71	365.98	0.66
**Random forest**	*Training*	554.82	288.7	0.47
	*Testing*	572.60	404.22	0.68
**XGBoost**	***Training***	**503.25**	**121.06**	**0.14**
	***Testing***	**208.00**	**83.32**	**0.13**

RMSE: Root Mean Square Error; MAE: Mean Absolute Error; MAPE: Mean Absolute Percentage Error; XGBoost: eXtreme Gradient Boosting.

### Risk factors examined by ML models

**Fig 6:**
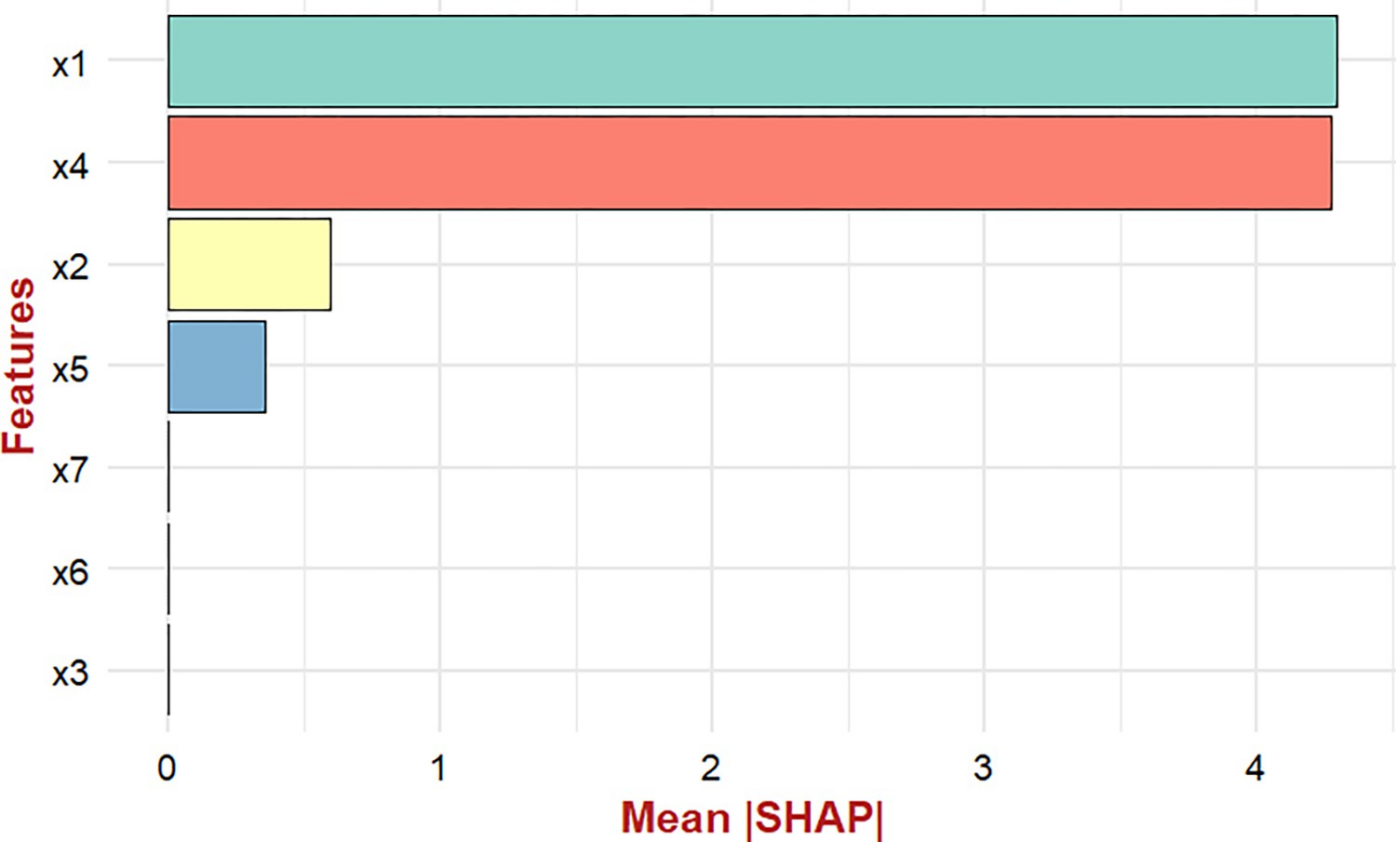
Important feature analysis of waterborne diseases by SHAP values; x1: mean temperature; x2: relative humidity; x3: maximum temperature; x4: minimum temperature; x5: precipitation; x6: maximum wind speed; x7: minimum wind speed.

Using the XGBoost model as the optimal choice, we identified crucial features for predicting waterborne diseases in Bangladesh using SHAP analysis. The analysis indicated that mean and minimum temperature were the primary determinants of waterborne diseases (S5 Table) while relative humidity, and precipitation were the tentative features of waterborne diseases in Bangladesh (Fig 6).

## Discussion

Waterborne diseases (WBDs) impose a significant health burden at the district level in Bangladesh. This study found that among the four WBDs analyzed, cholera was the most prevalent, with higher incidence rates observed in the districts of Chapai-Nawabganj, Brahmanbaria, Faridpur, Nilphamari, and Pirojpur followed by typhoid, amoebiasis and hepatitis A. Additionally, when considering overall WBDs incidence, the districts of Chapai-Nawabganj, Brahmanbaria, Faridpur, Nilphamari, and Chuadanga were identified as the most affected. This study further explored the key meteorological factors influencing the transmission of these diseases. The findings, while consistent with some prior research, diverged from others, underscoring the complex relationship between climate variables and disease spread. For example, mean temperature is significantly related to the transmission of cholera which aligns with a previous study conducted in Kolkata, India [64]. Similarly, mean temperature, precipitation and wind speed were significantly associated with the transmission of typhoid which aligns with a previous study conducted in Indonesia [65]. However, maximum temperature was negatively associated with hepatitis A which aligns with a previous study conducted in Korea [66]. Mean temperature showed a positive relationship with hepatitis A which contrasts with a previous study conducted in Korea [66]. Overall mean temperature was significantly associated with the transmission of waterborne diseases in Bangladesh.

The intricacies of these findings might be attributed to the diverse characteristics of waterborne diseases and the interactions among several factors. Waterborne disease transmission is often influenced by several nonlinear factors, posing challenges for traditional OLS regression or spatial regression methods to capture these nonlinear effects. However, these problems can efficiently be addressed by the ML models. In this study, we assessed the predictive performance of three tree-based ML models, e.g., decision tree (DT) model, random forest (RF) and ensemble extreme gradient boosting (XGBoost) for waterborne diseases using three prominent evaluation metrics such as root mean square error (RMSE), mean absolute error (MAE) and mean absolute percentage error (MAPE). Among these, the XGBoost model demonstrated superior performance in predicting waterborne diseases in Bangladesh, achieving a MAPE of just 0.13% which was the lowest among the models. Therefore, employing SHAP analysis based on the XGBoost model, we identified the primary risk factors that contribute the most to the transmission of waterborne diseases. The findings revealed that mean and minimum temperature, relative humidity and precipitation were the key determinants for the transmission of waterborne diseases in Bangladesh. A salient finding of our study was the discernible decline in the number of cases of WBDs in 2020. The COVID-19 pandemic, which resulted in extensive public health efforts including lockdowns, social distancing, and improved hygiene procedures, is most likely to blame for this anomaly. These actions probably stopped the spread of other infectious diseases, such as waterborne infections, in addition to curbing the spread of COVID-19.

The study’s findings show a relationship between the transmission of waterborne illnesses and climatic conditions. Insights into disease dynamics may be gained from the spatiotemporal distribution of waterborne illnesses and their correlation with climate factors. The diverse ways that climate conditions affect different diseases highlight how complicated these interactions are, pointing to a region-specific effect that is probably driven by subtle differences in the local climate. This emphasizes how crucial it is to place interactions with meteorological parameters within the particular climatic setting of each field of study. Our research highlights the necessity for a targeted approach to disease prevention and control at the district level and has significant implications for public health in Bangladesh. By applying three tree-based machine learning models—DT, RF, and XGBoost—we were able to forecast waterborne infections without relying on assumptions. These models were simple to integrate into common software applications. Specifically, the XGBoost model outperformed the others in terms of prediction accuracy, as demonstrated by its superior performance across several evaluation metrics. Due to its high predictive accuracy and SHAP analysis’s ability to identify important risk factors, our proposed model may prove to be a valuable resource for organizing early warning systems. Even though our research does not focus on seasonality or future forecasts, the model’s ability to pinpoint key climatic risk factors—such as temperature, precipitation, and humidity—can assist in proactive disease management by alerting decision-makers to periods of increased risk. This can help guide prompt actions and targeted preventive measures to control waterborne illness outbreaks in Bangladesh.

## Limitation

Although not all of these characteristics were found to be statistically significant, the study’s findings show that some climatic conditions are linked to the spread of waterborne infections. A key limitation is the availability of only four years’ worth of annual data, which restricted the number of disease data and the ability to control for confounders, and the measurement of seasonal effects. Additionally, factors known to influence waterborne infections, such as population density, air pressure, and air quality, were not included. The absence of detailed socioeconomic data also limits our understanding of the complex interactions between socioeconomic factors and the prevalence of waterborne illnesses. This underscores the need for future research to include socioeconomic elements and more comprehensive data to better elucidate these interactions.

## Conclusion

This study, especially in light of climate change, emphasizes the important role that temperature plays as a climatic risk factor for waterborne infections in Bangladesh. The results highlight the necessity of district-level public health plans that are customized to the unique geographic and climatic circumstances of the area. By using a One Health concept, this research offers valuable insights for district administration and local development activities. The findings underscore the significance of creating adaptable plans to lessen the effects of climate change on public health, particularly in vulnerable areas. To address these issues, we recommend bolstering climate-responsive health systems, raising community awareness of the dangers of waterborne illnesses, investing in essential infrastructure for access to clean water and sanitation, and supporting multidisciplinary research. Policymakers should incorporate these findings into climate adaptation plans to maintain the effectiveness of public health interventions in the face of environmental changes.

## Supporting information

S1 Fig Cross validation and parameter tuning of decision tree model for waterborne diseases. (TIF)

S2 Fig Cross validation and parameter tuning of random forest model for waterborne diseases. (TIF)

S3 Fig Learning curve of XGBoost model for waterborne diseases. (TIF)

S1 Table Incidence rates of different waterborne diseases in Bangladesh from 2017–2020. (XLSX)

S2 Table Descriptive statistics of yearly climate factors in Bangladesh from 2017 to 2020. (XLSX)

S3 Table Hyperparameters and cross-validation of XGBoost model. (XLSX)

S4 Table Pearson correlation matrix between different waterborne diseases and climate factors. (XLSX)

S5 Table Important features of infectious diseases by SHAP analysis for XGBoost model as optimal choice. (XLSX)

S1 Text Incidence rates of waterborne diseases. (DOCX)
