# Supplementary material for: Machine learning and spatio-temporal analysis of meteorological factors on waterborne diseases in Bangladesh
Source: PLoS Negl Trop Dis. 2025 Jan 16;19(1):e0012800. doi: 10.1371/journal.pntd.0012800 (PMC11737758; doi:10.1371/journal.pntd.0012800)
Supplement: S1 Text — (DOCX) [file pntd.0012800.s009.docx]

***Supporting Information***

**Machine learning and spatio-temporal analysis of meteorological factors on waterborne diseases in Bangladesh**

Arman Hossain Chowdhury^1^, Md. Siddikur Rahman^1*^

^1^Department of Statistics, Begum Rokeya University, Rangpur, Rangpur-5404, Bangladesh

### **Incidence rates of waterborne diseases**

The incidence rate is an epidemiological metric that quantifies the frequency of new illness or condition cases within a defined population during a specified timeframe.

We calculated the incidence rate by the following formula:

$Incidence rate=\frac{New cases}{Total population at risk}\times100,000$ (1)

Here, new cases denote the count of individuals who contract the disease or condition within the designated timeframe, while total population at risk encompasses all individuals in the population who are vulnerable to the disease. The factor of 100,000 is employed to present the rate in a standardized manner, facilitating comparisons across various populations or regions.
